# Polymorphisms of the *TGF-β1* gene and the risk of acquired aplastic anemia in a Chinese population

**DOI:** 10.1007/s00277-016-2886-5

**Published:** 2016-12-09

**Authors:** Xue-hong Liang, Liucheng Rong, Guangsheng He, Hailong He, Shengyun Lin, Yan Yang, Yao Xue, Yongjun Fang

**Affiliations:** 1 Department of Hematology and Oncology, Children’s Hospital of Nanjing Medical University, Nanjing, China; 2 Department of Hematology, Jiangsu Province Hospital/The First Affiliated Hospital of Nanjing Medical University, Nanjing, China; 3 Department of Hematology and Oncology, Soochow Children’s Hospital Affiliated to Soochow University, Suzhou, China; 4 Department of Hematology, First Affiliated Hospital, Zhejiang Chinese Medical University, Hangzhou, China; 5 Department of Hematology and Oncology, The First Hospital of Jilin University, Changchun, China

**Keywords:** Acquired AA, *TGF-β1* gene, Polymorphisms, Risk

## Abstract

Acquired aplastic anemia (AA) is a hematological disease characterized by failure of bone marrow hematopoiesis resulting in pancytopenia. While immune-mediated destruction of hematopoietic stem/progenitor cells (HSPCs) plays a central role in the pathophysiology of acquired AA, the transforming growth factor-β1 (TGF-β1) is crucial in adjusting the immune system. The aim of our study was to investigate the role of *TGF-β1* gene polymorphisms rs1800469 and rs2317130 in susceptibility to acquired AA. Via the approach of SNaPshot, we genotyped rs1800469 and rs2317130 in 101 patients with acquired AA and 165 controls. It derived us to the conclusion that the genotype *TT* of rs1800469 (*C/T*) was significantly associated with decreased risk of acquired AA (adjusted OR = 0.39, 95% CI = 0.18–0.83, *P* = 0.014). Furthermore, this decreased risk was more pronounced among male patients (adjusted OR = 0.35, 95% CI = 0.13–0.95, *P* = 0.038) and SAA/vSAA (severe AA/very severe AA) patients (adjusted OR = 0.31, 95% CI = 0.12–0.77, *P* = 0.02) compared with controls in subgroup analysis. However, a significant increased risk was observed in the genotype distributions of rs2317130 for *TT* genotype (adjusted OR = 2.52, 95% CI = 1.03–6.19, *P* = 0.04) compared with the *CC* genotype among the SAA/vSAA patients and controls in the severity stratification analysis. Our results indicated that *TGF-β1* gene polymorphisms might be involved in the munity of acquired AA in a Chinese population. This initial analysis provides valuable clues for further study of *TGF-β1* pathway genes in acquired AA.

## Introduction

Acquired aplastic anemia (AA) is a hematological disease with the failure of bone marrow hematopoiesis resulting in pancytopenia. Acquired AA is a rare disorder with about 1–2 new cases per million per year in western countries; however, the incidence of AA is about threefold higher in East Asia [1]. Until now, its actual cause remains unknown. Possible mechanisms responsible for acquired AA include immunologically mediated damage to the hematopoietic stem cells and abnormalities in the hematopoietic microenvironment [2].

Immune abnormalities play a critical role in acquired AA, especially T cell-mediated immune damage, which is closely associated with bone marrow failure [3]. Transforming growth factor-β1 (TGF-β1*)* is one of a variety of immune molecules expressed in the immune system [4]. The paramount function of TGF-β1 in the immune system is to regulate T lymphocyte responses, including proliferation, differentiation, and apoptosis [5]. Also, as some studies suggest, TGF-β1 seems to prevent hematopoietic stem cells (HSC) reentering into the cell cycle by upregulating transcription of the cyclin-dependent kinase (CDK) inhibitor p57Kip2 and suppressing PI3K/Akt signaling [6]. Due to the close relationship among TGF-β1 and the immune system and HSC, the abnormal expression of *TGF-β1* gene may be related to the pathogenesis of acquired AA. In humans, the *TGF-β1* gene is located on chromosome 19q13, and contains seven exons and six introns [7]. Recent studies indicate that *TGF-β1* single nucleotide polymorphisms (SNPs) mainly have relationship with diseases of the following categories: tumor diseases [8, 9], organ fibrosis [10], and autoimmune diseases [11]. To our knowledge, there exist few studies attempting to clarify the association between *TGF-β1* SNPs and acquired AA [12]. Thus, our study aims to investigate the role of *TGF-β1* gene polymorphisms rs1800469 and rs2317130 in susceptibility to acquired AA in a Chinese population.

## Materials and methods

### Study subjects

All patients were recruited in five hospitals (Children’s Hospital of Nanjing Medical University, Soochow Children’s Hospital Affiliated to Soochow University, The First Hospital of Jilin University, The First Affiliated Hospital of Zhejiang Chinese Medical University, and Jiangsu Province Hospital/The First Affiliated Hospital of Nanjing Medical University) between July 2014 and December 2015. All subjects were genetically unrelated individuals of Han Chinese descent. The research protocol was approved by the Medical Ethics Committee of Children’s Hospital of Nanjing Medical University. Written informed consent was obtained from the parents or legal guardians of all the participation.

The study included 101 acquired AA patients and 165 age- and sex-matched healthy controls. All patients were diagnosed with acquired AA by standard procedures [1]. The AA diagnosis and severity stratification (Table 1) of the cases were determined uniformly according to the Suggestion of Diagnosis and Treatment of Acquired Aplastic Anemia in Childhood, published by the Society of Pediatrics, Chinese Medical Association in 2014. Bone marrow biopsy evaluation had been done in all patients. All patients were studied to rule out infections, hypoplastic myelodysplasia/leukemia, paroxysmal nocturnal hemoglobinuria (PNH), and congenital marrow failure syndromes, including Fanconi anemia, dyskeratosis congenita, thrombocytopenia absent radius syndrome, Diamond-Blackfan anemia, Shwachman-Diamond syndrome, and severe congenital neutropenia [13, 14]. PNH clones were detected in patients with acquired AA. PNH clones were confirmed with peripheral blood flow cytometry to detect the absence or severe deficiency of glycosyl phosphatidylinositol-anchored proteins (GPI-APs) on two lineages. The clone size was evaluated in neutrophil after staining cells with monoclonal antibodies and a reagent known as fluorescent aerolysin (FLAER). The laboratory cutoff for PNH clone was established at 1%. The control subjects were healthy individuals and randomly selected following health examination at the same time, in the same geographic areas, without malignant neoplasms, autoimmune diseases, or other blood system disease.

**Table 1:** The severity of AA is defined according the modified Camitta criteria

Non-severe AA	Decreased bone marrow cellularity and peripheral blood cytopenia, not fulfilling criteria for SAA
Severe AA	Bone marrow cellularity <25%
	At least 2 of
	neutrophil count <0.5 × 10^9^/l
	platelet count <20 × 10^9^/l
	reticulocyte count <20 × 10^9^/l
Very severe AA	Fulfilling criteria of SAA plus neutrophil count <0.2 × 10^9^/l

Each case and control subject provided a 2–3-mL venous blood. The anticoagulation tubes with ethylenediaminetetraacetic acid were used as blood sample storages. DNA was extracted using the TIANamp blood DNA kit (Tiangen Biotech, Beijing, China) according to the manufacturer’s instructions.

### SNP selection and genotyping

Han Chinese date in HapMap database (HapMap Data Rel 24/phaseII, Nov08, on NCBI B36 assembly, dbSNP b126) was used when we selected the tSNPs of *TGF-β1* gene. The tSNPs had a minor allele frequency (MAF) > 5% within the *TGF-β1* gene region (including 2-kb upstream and 2-kb downstream) using the pairwise option of the Haploview 4.0 software, and *r* ^*2*^ *= 0.8* was selected as a threshold for the analyses. Finally, in the present study, two tSNPs (rs1800469 and rs2317130) were selected.

The genotype was done using the SNaPshot genotyping approach (Nanjing Biohelper tech center). For confirmation of the genotype, approximately 10% of samples were randomly selected for repeat analysis, and the results were 100% concordant.

### Statistical analysis

Hardy-Weinberg equilibrium of the genotype distribution among the controls was evaluated by a goodness-of-fit χ^2^ test to identify possible selection genotyping errors and bias. Nonparametric Mann-Whitney test was used for analyzing the distribution differences of age between the cases and controls. Chi-square (χ^2^) test was used to estimate the distribution differences of gender as well as each genotype of rs1800469 and rs2317130 between the two groups. Unconditional multivariate logistic regression analyses were performed to obtain crude and adjusted odds ratios (ORs) for risk of acquired AA and their 95% confidence intervals (CIs) with adjustment for diagnosis age and gender. Two-sided *P* values were selected, and *P* < 0.05 was set as the threshold for statistical significance. Haploview version 4.0 was used to calculate the D’ value and r2 value among the two SNPs. All statistical analyses were performed using Statistics Analysis System software (version 9.2; SAS Institute, Cary, NC).

## Result

### Characteristics of the study subjects

The demographic characteristics of the study subjects are summarized in Table 2. There was no significant difference in the distribution of age (*P* = 0.234) between cases (median age 21 years, range 1–87 years) and controls (median age 25 years, range 1–87 years). It showed no statistical difference in gender (*P* = 0.441) between two groups. Twenty-four NSAA (non severe AA) and 69 SAA/vSAA (severe AA/very severe AA) were included in patients. In addition, six (5.94%) out of 101 patients had PNH clone and all the clone size were below 50%.

**Table 2:** Distribution of selected variables in Chinese-acquired AA cases and controls

Variables	Cases(*n* = 101)		Controls (*n* = 165)		P*
	n	%	n	%	
Age					
≦16	44	43.56	64	38.79	0.234^a^
>16	57	56.44	101	61.21	
Gender					
Male	57	56.44	100	60.61	0.441^b^
Female	44	43.56	65	39.39	
Severity branch					
NSAA	24	23.76			
SAA/vSAA	69	68.32			
Missing	8	7.92			
PNH clone					
PNH+	6	5.94%			
PNH−	95				

PNH+, PNH clone size > 1%; PNH−, PNH clone size not > 1%

*NSAA* non-severe AA, *SAA* severe AA, *vSAA* very severe AA, *PNH* paroxysmal nocturnal hemoglobinuria

^a^Mann-Whitney test

^b^Chi-square (×2) test

### Association between *TGF-β1* polymorphisms and acquired AA risk

The genotype frequencies of these two polymorphisms among the controls were all in agreement with Hardy–Weinberg equilibrium. The multivariate logistic regression analysis was done to evaluate the association between *TGF-β1* gene rs1800469 and rs2317130 polymorphisms and acquired AA (shown in Table 3). It revealed a protective effect in homozygous *TT* genotype of rs1800469 (adjusted OR = 0.39, 95% CI = 0.18–0.83, *P* = 0.014) compared with the wild-type *CC*. We also observed a lower risk in the recessive model of rs1800469 polymorphisms (adjusted OR = 0.50, 95% CI = 0.27–0.96, *P* = 0.036), while not in the dominant model (adjusted OR = 0.58, 95% CI = 0.33–1.03, *P* = 0.064). However, no significant association had been found between polymorphisms of rs2317130 and susceptibility for acquired AA.

**Table 3:** Genotype frequencies of the Rs1800469 and Rs2317130polymorphism among acquired AA cases and controls and associations with acquired AA risk

Genotype	Cases(*n* = 101)		Controls(*n* = 165)		OR(95%CI)	Adjusted OR(95%CI)^a^	Adjusted *P* ^a^ *value*
	*n*	%	*n*	%			
Rs1800469 C/T							
CC	30	29.70	33	20.00	1.00(reference)	1.00(reference)	0.056
CT	55	54.46	88	53.33	0.69(0.38–1.25)	0.68(0.37–1.24)	0.204
TT	16	15.84	44	26.67	0.40(0.19–0.85)	0.39(0.18–0.83)	0.014*
TT + CT	71	70.30	132	80.00	0.59(0.33–1.049)	0.58(0.33–1.03)	0.064
CC + CT	85	84.16	121	73.33	1.00(reference)	1.00(reference)	
TT	16	15.84	44	26.67	0.52( 0.27–0.98)	0.50(0.27–0.96)	0.036*
Rs2317130 C/T							
CC	17	16.83	41	24.85	1.00(reference)	1.00(reference)	0.191
CT	55	54.46	89	53.94	1.49(0.77–2.88)	1.54(0.79–2.98)	0.204
TT	29	28.71	35	21.21	1.99(0.94–4.23)	2.10(0.99–4.49)	0.054
TT + CT	84	83.17	124	75.15	1.63(0.87–3.07)	1.69(0.89–3.19)	0.105
CC + CT	72	71.29	130	78.79	1.00(reference)	1.00(reference)	
TT	29	28.71	35	21.21	1.49(0.84–2.64)	1.54(0.87–2.73)	0.142

*Means *P* < 0.05

^a^Logistic regression analysis was adjusted for age and gender

### The stratified analysis of the associations between *TGF-β1* polymorphisms and clinical features of acquired AA

The age and gender stratification analyses are shown in Table 4. The *TT* genotype of rs1800469 develop a protective role (adjusted OR = 0.35, 95% CI = 0.13–0.95, *P* = 0.038) compared with the *CC* genotype in male patients, but not in females. On the contrary, no significant association was observed between the genotypes of rs1800469 and risk of acquired AA in the age stratification analysis. However, no significant association had been found between polymorphisms of rs2317130 and susceptibility for acquired AA in the age and gender stratification analyses.

**Table 4:** Association between the Rs1800469 and Rs2317130 polymorphism among gender and age of acquired AA patients

	Case *n* (%)	Control *n* (%)			Adjusted OR (95% CI)^a^			
			CC	CT	TT	CT/TT	CC/CT	TT
Rs1800469C/T gender								
Male	57(56.44)	100(60.61)	1.00 (reference)	0.58(0.27–1.28)	0.35(0.13–0.95)*	0.51(0.24–1.07)	1.00 (reference)	0.51(0.22–1.17)
Female	44(43.56)	65(39.39)	1.00 (reference)	0.86(0.34–2.19)	0.47(0.15–1.53)	0.73(0.30–1.78)	1.00 (reference)	0.52(0.20–1.40)
Age								
<=16	44(43.56)	64(38.79)	1.00 (reference)	0.61(0.24–1.57)	0.43(0.14–1.37)	0.55(0.22–1.36)	1.00 (reference)	0.61(0.24–1.57)
>16	57(56.44)	101(61.21)	1.00 (reference)	0.75(0.34–1.62)	0.37(0.13–1.01)	0.62(0.29–1.30)	1.00 (reference)	0.45(0.19–1.07)
Rs2317130 C/T gender								
Male	57(56.44)	100(60.61)	1.00 (reference)	1.60(0.67–3.87)	2.61(0.97–7.04)	1.88(0.81–4.35)	1.00 (reference)	1.86(0.88–3.91)
Female	44(43.56)	65(39.39)	1.00 (reference)	1.40(0.51–3.83)	1.44(0.45–4.63)	1.41(0.54–3.71)	1.00 (reference)	1.13(0.46–2.76)
Age								
<=16	44(43.56)	64(38.79)	1.00 (reference)	1.26(0.48–3.31)	1.92(0.61–5.99)	1.43(0.57–3.59)	1.00 (reference)	1.63(0.65–4.07)
>16	57(56.44)	101(61.21)	1.00 (reference)	1.77(0.71–4.41)	2.21(0.80–6.11)	1.90(0.79–4.58)	1.00 (reference)	1.44(0.69–3.00)

*Means adjusted *P* = 0.038

^a^Logistic regression analysis was adjusted for age and gender

The severity stratification analysis was presented in Tables 5, and subjects with *TT* genotype of rs1800469 had a significantly lower risk of AA (adjusted OR = 0.31, 95% CI = 0.12–0.77, *P* = 0.02) compared with the *CC* genotype in the SAA/vSAA patients. Similarly, in the recessive model, the *TT* genotype of rs1800469 decreased the incidence of acquired AA (adjusted OR = 0.34, 95% CI = 0.18–0.88, *P* = 0.02) in the SAA/vSAA patients. On the contrary, a significant increased risk was observed for *TT* genotype of rs2317130 (adjusted OR = 2.52, 95% CI = 1.03–6.19, *P* = 0.04) compared with the *CC* genotype in the SAA/vSAA patients.

**Table 5:** Association between the Rs1800469 and Rs2317130 polymorphism and the risk branch of acquired AA patients

Genotype	control *n* (%)	Clinical risk *n* (%)		OR (95%CI)		Adjusted OR (95% CI)^a^	
		NSAA	SAA/vSAA	NSAA	SAA/VSAA	NSAA	SAA/vSAA
Rs1800469C/T							
CC	33(20.0)	6(25.0)	21(30.4)	1.00 (reference)	1.00 (reference)	1.00(reference)	1.00 (reference)
CT	88(53.3)	11(45.8)	39(56.5)	0.69(0.23–2.01)	0.69(0.36–1.35)	0.72(0.24–2.11)	0.69(0.35–1.35)
TT	44(26.7)	7(29.2)	9(13.0)	0.87(0.27–2.85)	0.32(0.13–0.79)	0.98(0.29–3.25)	0.31(0.12–0.77)*
TT + CT	132(80)	18(75.0)	48(69.5)	0.75(0.28–2.04)	0.57(0.30–1.08)	0.80(0.29–2.20)	0.56(0.29–1.07)
CC + CT	121(73.3)	17(70.8)	60(86.9)	1.00 (reference)	1.00 (reference)	1.00 (reference)	1.00 (reference)
TT	44(26.7)	7(29.2)	9(13.0)	1.13(0.44–2.92)	0.41(0.19–0.90)	1.23(0.47–3.22)	0.34(0.18–0.88)**
Rs2317130 C/T							
CC	41(24.9)	7(29.2)	10(14.5)	1.00 (reference)	1.00 (reference)	1.00 (reference)	1.00 (reference)
CT	89(53.9)	11(45.8)	39(56.5)	0.72(0.26–2.00)	1.80(0.82–3.95)	0.67(0.24–1.87)	1.874(0.85–4.16)
TT	35(21.2)	6(25.0)	20(29.0)	1.00(0.31–3.27)	2.34(0.97–5.67)	0.88(0.26–2.93)	2.52(1.03–6.19)***
TT + CT	124(75.2)	17(70.8)	59(85.5)	0.80(0.31–2.07)	1.95(0.92–4.16)	0.73(0.28–1.91)	2.05(0.95–4.42)
CC + CT	130(78.8)	18(75.0)	49(71.0)	1.00 (reference)	1.00 (reference)	1.00 (reference)	1.00 (reference)
TT	35(21.2)	6(25.0)	20(29.0)	1.24(0.46–3.35)	1.52(0.80–2.88)	1.15(0.42–3.16)	1.58(0.82–3.03)

*Means adjusted *P* = 0.02

**Means adjusted *P* = 0.02

***Means adjusted *P* = 0.04

^a^Logistic regression analysis was adjusted for age and gender

## Discussion

Acquired AA is a life-threatening disorder, characterized by the failure of bone marrow hematopoiesis without dysplasia or fibrosis and peripheral blood pancytopenia. Immune-mediated destruction is important among the causes of acquired AA [2]. The abnormal immunity in acquired AA mainly includes the over-function of T lymphocytes involving Th1 cells and CD8+ T cells, the deficient immune regulation of CD4+CD25+ T regulatory cells, NKT cells, monocytes, and decreased NK cells [15], while TGF-β1 plays a critical role in the regulation of immune responses. As the first observation of TGF-β1 regulation of immune cell functions was made three decades ago [16] , much progress has been made in the knowledge of TGF-β1 in regulating immune cell function [17]: TGF-β1 blocks proliferation of T cell by inhibiting IL-2 production and inhibits Th1 and Th2 cells differentiation through downregulating the function or expression of T-bet/Stat4 and GATA-3/NFAT; also, TGF-β1 inhibits cytotoxic T lymphocytes (CTL) differentiation and T cell activation-induced cell death (AICD); in addition, TGF-β1 plays a role in inhibiting NK cell functions through attenuating IFN-γ production and its cytolytic activity [5]. Meanwhile, TGF-β1 has been described as an important regulator of hemopoiesis [18]. The effect of TGF-β1 is biphasic in HSCs: at high levels, *TGF-β1* inhibits proliferation of both myeloid-biased (My-) and lymphoid-biased (Ly-) HSCs; at lower levels, however, TGF-β1 stimulates My-HSC and impairs Ly-HSC proliferation in vitro [19, 20]. So we speculate that TGF-β1 may have a relationship with acquired AA. In this study, we investigate the influence of *TGF-β1* polymorphisms on the risk of developing acquired AA in a Chinese population.

Our study showed that the *C* to *T* variation of rs1800469 was associated with lower risk for acquired AA, especially in the SAA/vSAA patients. On the contrary, the *TT* genotype of rs2317130 showed increasing risk only in the SAA/vSAA patients compared to *CC*. To sum up, our results highlight that *TGF-β1* polymorphisms may play a role in the risk of acquired AA.

Past studies have shown that the level of TGF-β1 has a relationship with acquired AA incidence. Rizzo et al. demonstrated that aplastic anemia is associated with a decreased TGF-β1 expression in peripheral blood circulation [21]. Similarly, a research on Egyptian shows that decreased TGF-β1, by acting as an inhibitor of T cells, leads to the underlying T cell-mediated marrow destruction in AA [22]. Thus, we speculate *TGF-β1* variants may relate to altering the expression of *TGF-β1*. However, the molecular mechanisms require further research.

Moreover, we found the *C* to *T* variation of rs1800469 is associated with a lower risk in males. As a rule, gender-specific effects of autosomal genes can be explained by epistasis with sex-linked genes or by interaction with nongenetic factors which are correlated with sex [23]. For TGF-β1 acted as one of androgen receptor-responsive target genes [24], the male-specific effect found in this study may be explained by TGF-1 dependent on androgen level.

Some limits of this study should be addressed. Firstly, our study is a hospital-based case-control design, which may lead to selection bias of subjects related with some particular genotypes. So we adopt rigorous exclusion criteria in recruitment of case and control subjects, which were matched by age, gender, and ethnicity, minimizing the potential confounding bias. Secondly, due to the limited samples, these results must be verified by further studies with larger patient populations. Last but not least, this study is restricted on the statistics level, and further functional research should be done to validate our findings and disclose the underlying molecular mechanisms.

In conclusion, our results suggest that *TGF-β1* polymorphisms may play an important role in the risk of acquired AA in a Chinese population. Detection of polymorphisms of *TGF-β1* may offer a new approach in the diagnosis and treatment of acquired AA. However, additional studies with larger sample sizes are needed to verify these findings and shed more light on the mechanisms of how *TGF-β1* gene act in patients with acquired AA.
